# The impact of posttraumatic stress disorder on the symptomatology of borderline personality disorder

**DOI:** 10.1186/s40479-016-0042-4

**Published:** 2016-08-01

**Authors:** Sylvia Cackowski, Tamar Neubauer, Nikolaus Kleindienst

**Affiliations:** 1 Department of Psychosomatic Medicine and Psychotherapy, Central Institute of Mental Health, Medical Faculty Mannheim, Heidelberg University, Mannheim, Germany; 2 Institute of Psychiatric and Psychosomatic Psychotherapy, Central Institute of Mental Health, Medical Faculty Mannheim, Heidelberg University, Mannheim, Germany

**Keywords:** Borderline personality disorder, Posttraumatic stress disorder, Childhood sexual abuse, Comorbidity

## Abstract

**Background:**

Previous findings on the impact of co-occurring posttraumatic stress disorder (PTSD) in patients with borderline personality disorder (BPD) have revealed inconsistencies, which may have been related to small sample sizes or differences in the presence of childhood sexual abuse (CSA). In this study, the potentially aggravating impact of PTSD and the role of CSA were examined in a large cohort of BPD patients.

**Methods:**

BPD patients with current PTSD (*n* = 142) were compared to BPD patients without PTSD (*n* = 225) regarding different BPD features such as non-suicidal self-injury. Further, we examined the potentially confounding role of CSA.

**Results:**

BPD patients with PTSD showed elevated affect dysregulation, intrusions, dissociation, history of suicide attempts and self-mutilation compared to those with only BPD. The effects of PTSD on BPD patients regarding dissociation and the history of suicide attempts were at least partially related to CSA.

**Conclusions:**

The additional diagnosis of PTSD in BPD patients can aggravate some, but not all BPD features. With respect to dissociation and suicide attempts, at least some of the impact seems to relate to CSA.

## Background

Borderline personality disorder (BPD) shows high comorbidity rates with several psychiatric disorders [1–4]. The rates of co-occurring posttraumatic stress disorder (PTSD) within samples of BPD patients range between 30 % and 79 %, with somewhat higher rates of comorbidity in clinical populations as compared to field-studies [3–7]. However, findings on the impact of additional PTSD on symptomatology in BPD patients remain inconsistent. Several studies revealed that women with BPD and co-occurring PTSD had worse general functioning, more severe BPD symptomatology [8–10], more frequent hospitalizations [8, 9, 11], increased dissociation [9], suicidality and impulsivity [8, 12], as well as increased self-mutilating behaviour [13, 14]. In a sample of 94 BPD patients, Harned and colleagues found higher rates of self-injury and suicide attempts based on interpersonal reasons in BPD patients with co-occurring PTSD, as well as more pronounced emotion dysregulation [13]. However, there were no differences found in the number of met BPD criteria and general psychosocial functioning between BPD patients with or without PTSD. In a longitudinal study by Gunderson and colleagues, co-occurring PTSD in BPD patients was associated with poorer 2-year outcomes based on the Global Assessment of Functioning Scale (GAF) [15, 16]. Furthermore, a 10-year follow-up study showed that the likelihood of symptom remission in BPD patients was lower in those with co-occurring PTSD [17]. Previous studies also revealed that the reported extent of sexual [4, 8, 13], physical, verbal, and emotional abuse [3, 8] was higher in patients with a combined diagnosis of BPD and PTSD. Nonetheless, several other studies did not find any significant impact of co-occurring PTSD on BPD symptom severity [11, 13, 18], suicidality, hospitalizations [13], or physical, emotional and sexual abuse severity [9].

Independent of ones BPD or PTDS diagnosis, childhood sexual abuse (CSA) has been found to relate to serious aspects such as self-mutilation, suicidality and dissociation [19, 20]. Although some controversial results have pointed out that CSA is neither a necessary nor sufficient factor in the development of BPD [19, 21–23], CSA has been intensely discussed as an etiological factor for BPD [14, 18]. Several studies have shown that the severity of CSA predicts the severity of BPD symptomatology [2, 3, 21, 24, 25]. It has been hypothesized that CSA may explain the presence and impact of PTSD in BPD patients, i.e., that CSA, rather than a PTSD diagnosis, might be predictive of self-mutilating behaviour, as well as more severe symptomatology in patients with BPD [13, 18].

Previous research addressing the relationship between BPD, PTSD and CSA has some noteworthy limitations. As Nepon and colleagues [26] noticed, samples of highly symptomatic treatment seeking patients, as studied by Harned and colleagues [13], may not be representative of a broader BPD population. On the other hand, it should also be noted that research on treatment seeking patients is particularly relevant for therapists, who seek information about the complexity of conditions of their clientele. Furthermore there is the assumption that discrepancies between the results of Harned and colleagues [13] and other (epidemiologic) studies [4] might be a consequence of different sample sizes. Although the patients investigated in our study were also treatment seeking, we aimed to overcome the limitation of a small sample size. To further enhance representability of our sample, no specific inclusion criteria were stated concerning the presence or frequency of self-injury and suicide attempts.

We aimed to investigate a large clinical sample of BPD patients divided in subgroups of patients with versus without co-occurring PTSD, to clarify whether additional PTSD is related to major aspects of BPD symptomatology such as the number of fulfilled BPD criteria, as well as suicidal and self-harming behaviour. In a next step, we analysed the impact of CSA. This seems important, as CSA affects BPD pathology [2, 3, 21], but does not necessarily lead to a PTSD diagnosis [27]. For instance, we expect CSA (rather than PTSD) to be predictive of self-mutilating behaviour and general symptom severity [13, 18].

## Methods

### Sample

Participants were treatment-seeking patients, mostly for a presumed diagnosis of BPD. Criteria for inclusion in the study were female gender, age between 18–65 years and a diagnosis of BPD according to DSM-IV [28]. Participants were excluded from the study if they met life-time criteria for schizophrenia, bipolar-I disorder, or mental disorder due to brain damage. All diagnostic and observer based assessment instruments were conducted by trained psychologists and physicians. BPD diagnosis (according to DSM-IV criteria) was determined by using the International Personality Disorder Examination (IPDE) [29]. Apart from the number of met BPD criteria, a dimensional IPDE score was calculated. Axis-I diagnoses, including PTSD were assessed using the Structural Clinical Interview for DSM-IV (SCID-I) [15]. Besides assessing whether the diagnostic criteria for PTSD was currently met (current PTSD), we also assessed whether PTSD criteria was met in the past. Up to 84 % of the patients with lifetime PTSD met the criteria for a current PTSD, therefore analyses were based on the current PTSD diagnosis, which was free of missing values (the results were almost identical when based on current or lifetime PTSD). A total of 722 female patients were assessed for eligibility, whereby 355 (49.2 %) of them were not included in the study, as not all of the predefined criteria for participation in the study were met. All of the remaining 367 female patients were included in the study.

### Assessment of clinical characteristics

To quantify borderline-typical symptomatology, the Borderline Symptoms List- 95 (BSL) [30] was applied, which is a self-report scale consisting of seven subscales (self-perception, affect regulation, auto aggression, dysphoria, isolation, intrusions, and hostility) and a general severity score of BPD symptomatology. The psychometric criteria of the BSL have been investigated in several studies and have demonstrated good reliability and validity [30]. Further clinical assessments comprised questionnaires on trait dissociation measured by the Dissociative Experiences Scale (DES) [31], childhood trauma history measured by Childhood Trauma-Questionnaire (CTQ) [32] and an assessment of the presence and frequency of previous suicide attempts, as well as the history of self-mutilation (“Have you ever had a suicide attempt in your life? How many suicide attempts did you have in your life? Have you ever used self-injuring behaviour?”). Participants were informed about the definition of self-injuring behaviour, which was determined as “any deliberate and self-inflicted behaviour with the intention to harm or destroy body tissue without suicidal intent”.

### Procedure

The current study is part of an observational study which was conducted from April 2001 to October 2007 at two German Departments of Psychiatry specialized in treatment of BPD: the Department of Psychiatry of the University in Freiburg and the Central Institute of Mental Health in Mannheim. The patients included in the study were treatment-seeking patients, mostly for a presumed diagnosis of BPD. Definite BPD diagnosis (according to DSM-IV; [28]) was determined by use of the International Personality Disorder Examination (IPDE; [29]). Axis-I diagnoses were established by use of the structural clinical interview for DSM-IV (SCID-I; [15]). The following inclusion criteria were required for study entry:

- female gender
- age ranging from 18 to 65
- established diagnosis of BPD (according to DSM-IV) lifetime
- written informed consent.

Patients with a lifetime diagnosis of schizophrenia, bipolar I disorder, or mental disorders due to brain damage were not included in this study. The investigation was conducted in accordance with GCP guidelines and the declaration of Helsinki and was approved by the Ethics committee of the Medical Faculty of Heidelberg University. The procedure of the study was explained in detail and a written informed consent was signed by all participants. Data on the potentially aggravating impact of attention-deficit hyperactivity disorder for BPD have been published elsewhere [33].

### Statistical analysis

Regression models were used to test whether a diagnosis of current PTSD was related to continuous data (such as DES total score). For modelling, binary data logistic regression was applied. Chi-square tests were used to compare categorical variables such as education, employment status and marital status. To compare ordinal data, such as years of schooling across groups, the Kolmogorov-Smirnoff test was used. We indicated with an asterisk which variables would stay significant on the alpha level of 0.05 (2-tailed) after Bonferroni correction.

To examine whether the relation between PTSD and BPD psychopathology (BSL, DES, suicidal and self-harming behaviour) was associated with CSA (measured by CTQ), we applied a mediational model as described by Baron and Kenny [34]. When these analyses referred to continuous dependent variables, regression coefficient relating PTSD and BPD-psychopathology was decomposed into two additive components: (a) the indirect effect related to CSA, and (b) the regression coefficient after controlling for CSA. These two components enabled us to determine to which extent the connection between PTSD and a dependent variable (BSL, DES, suicidal and self-harming behaviour) was associated with CSA [35]. As pointed out by Preacher and Hayes [36], the formal evaluation of statistical significance of the indirect effect should be based on non-parametric methods, as the sampling distribution of the indirect effect is typically skewed. This skewedness violates the assumption of normality, which is at the base of e.g., the Sobel-test. We followed the recommendation by Hayes [37] and estimated the standard error of the parameter corresponding to the indirect effect from bootstrapping using 10,000 bootstrapping samples for each analysis by applying the macro PROCESS realized by Hayes in SPSS [37]. The parameters corresponding to the indirect effect were considered significant if the 95 % confidence interval around the point estimate of the parameter did not include zero.

## Results

### Demographic and clinical characteristics

Of the 367 BPD patients, 142 (38.7 %) met the criteria for current PTSD. Another 27 patients met the criteria for lifetime PTSD but not current PTSD. The most commonly reported traumatic events were^1^: sexual abuse or harassment (76 %), physical violence (35 %), witness of traumatic events such as violence against or abuse of others (28 %), accidents (7 %) and raid or threat with a weapon (5 %). More than one trauma type was experienced by 54 % of these patients.

Patients with and without PTSD did not differ significantly on any demographic characteristics (see Table 1). However, self-reported CSA occurred more frequently in the subgroup of BPD patients with a co-occurring diagnosis of PTSD than in BPD patients without co-occurring PTSD (81.2 % vs. 53.1 %, *p* ≤ 0.01). The correlation between self-reported CSA and a diagnosis of PTSD was 0.429 (*p* ≤ 0.01). Further clinical characteristics of BPD patients with and without PTSD and statistical group comparisons are depicted in Table 2.

**Table 1:** Demographic characteristics of BPD patients with and without PTSD

	Total		PTSD absent		PTSD present		P
	N	%	N	%	N	%	
Age (M ± SD)	28.6 ± 7.9		28.8 ± 7.9		28.1 ± 7.9		0.46
Schooling							0.08
No schooling	5	1.5	3	1.5	2	1.5	
9 years of schooling	73	21.4	42	20.6	31	22.6	
10 years of schooling	150	44.0	80	39.2	70	51.1	
12–13 years of schooling	113	33.1	79	38.7	34	24.8	
Education							0.13
Apprenticeship, University	205	61.0	128	64.3	77	56.2	
No degree	131	39.0	71	35.7	60	43.8	
Employment status							0.48
Employed	86	25.2	54	26.6	32	23.2	
Unemployed	255	74.8	149	73.4	106	76.8	
Marital status							0.41
Single, divorced/widowed	273	82.2	164	83.7	109	80.1	
Married	59	17.8	32	16.3	27	19.9	

Valid numbers and percentages (i.e., not including missing data) are presented

**Table 2:** Clinical characteristics of BPD patients with and without PTSD

	Total	PTSD absent	PTSD present	P
	M +/- SD	M +/- SD	M +/- SD	
CTQ				
History of CSA	63.3 %	53.1 %	81.2 %	≤0.01
DES	29.74 +/- 16.19	27.37 +/- 15.37	33.47 +/- 16.81	≤0.01
Suicide attempts/self-mutilation				
Frequency of suicide attempts	4.14 +/- 7.41	4.26 +/- 8.50	3.98 +/- 5.51	0.90
History of suicide attempts	71.92 %	66.8 %	79.5 %	0.02
History of self-mutilation	95.02 %	92.7 %	98.4 %	0.03
BSL				
BPD severity	206.41 +/- 68.30	201.03 +/- 70.87	215.12 +/- 63.23	0.07
Self-perception	35.65 +/- 17.83	34.42 +/- 18.50	37.64 +/- 16.57	0.12
Affect regulation	32.59 +/- 10.45	31.68 +/- 10.98	34.09 +/- 9.39	0.04
Auto aggression	28.69 +/- 13.07	27.77 +/- 13.47	30.17 +/- 12.32	0.11
Dysphoria	32.65 +/- 5.94	32.58 +/- 6.24	32.77 +/- 5.44	0.78
Isolation	23.57 +/- 10.51	23.36 +/- 10.59	23.90 +/- 10.40	0.65
Intrusions	13.86 +/- 8.19	12.49 +/- 8.11	16.05 +/- 7.86	≤0.001
Hostility	10.15 +/- 5.22	10.04 +/- 5.35	10.32 +/- 5.03	0.64
IPDE				
Number of BPD criteria	6.63 +/- 1.24	6.55 +/- 2.02	6.76 +/- 1.19	0.90
BPD dimensional score	14.49 +/- 1.97	14.39 +/- 1.26	14.66 +/- 1.88	0.21

### The role of CSA

As shown in the Table 3, dissociation as assessed by the DES total scores was significantly related to the diagnosis of PTSD (*p* = 0.02). When one separates the regression coefficient (b = 5.40) into an indirect component related to CSA (b = 4.16, p ≤ 0.01) and the direct component after controlling for CSA (b = 1.24, *p* = 0.62), the results suggest that the relationship between the diagnosis of PTSD and higher DES scores is essentially related to the presence of CSA (*p* ≤ 0.01) and is no longer significant after controlling for CSA. Similarly, we detected a significant relationship between PTSD and the occurrence of at least one suicide attempt (*p* ≤ 0.01). In percentages, 79.5 % of BPD patients with co-occurring PTSD tried to commit suicide, compared to 66.8 % of BPD patients without PTSD. CSA significantly affected the link between PTSD and the history of suicide attempts in BPD patients, as this link became non-significant after controlling for CSA (*p* = 0.23).

**Table 3:** Indirect effects related to CSA

	Total effect of PTSD	Indirect component related to CSA	Direct component after controlling for CSA
	Regression coefficients, SEs and *P*-values	Regression coefficients, SEs and *P*-values	Regression coefficients, SEs and *P*-values
DES	5.40 ± 2.32 (*p* = 0.02)	4.16 ± 1.38 (*p* ≤ 0.01)*	1.24 ± 2.51 (*p* = 0.62)
Suicide attempts/self-mutilation			
History of suicide attempts**	0.83 ± 0.31 (*p* ≤ 0.01)*	0.51 ± 0.19 (*p* ≤ 0.01)*	0.40 ± 0.33 (*p* = 0.23)
BSL			
Affect regulation	3.53 ± 1.40 (*p* = 0.01)	1.11 ± 0.63 (*p* = 0.08)	2.42 ± 1.54 (*p* = 0.12)
Intrusions	3.25 ± 1.04 (*p* ≤ 0.01)	1.25 ± 0.56 (*p* = 0.02)	2.00 ± 1.16 (*p* = 0.09)

^*^Remains statistically significant after Bonferroni-correction

^**^Remains statistically significant when considering lifetime PTSD (*p* ≤ 0.01)*

As the (lifetime) history of at least one suicide attempt might be related to lifetime PTSD rather than to current PTSD, analyses were also carried out for lifetime PTSD. Hereby, the results were fully confirmed. In contrast to the results on the occurrence of at least one suicide attempt, the frequency of previous suicide attempts was not significantly related to the diagnosis of PTSD (*p* = 0.90). Concerning the history of self-mutilation, the relation with PTSD diagnosis showed a trend (*p* = 0.09). 98.4 % of BPD patients with PTSD self-mutilated compared to 92.7 % of BPD patients without PTSD. There was no significant effect of CSA on self-mutilation and the connection stayed non-significant after controlling for CSA (*p* = 0.10). As further shown in Table 3, two of the seven BSL subscales showed a significant relation with PTSD diagnosis: intrusions (*p* ≤ 0.01) and affect regulation (*p* = 0.01).

## Discussion

This study adds to a growing body of evidence about patients with BPD and co-occurring PTSD. According to our results, PTSD is significantly related to dissociation (DES) and a history of suicide attempts in BPD patients. A trend was found for the impact of PTSD on the symptom severity (BSL) and the history of self-mutilation in patients with BPD.

Our finding that patients with co-occurring current PTSD scored significantly higher on the DES than patients without a co-occurring diagnosis, is in line with the findings from Heffernan and Cloitre, who also reported higher scores on the DES in the group of BPD patients with co-occurring PTSD [9]. However, this association became non-significant in our study after controlling for CSA, suggesting that the effect of PTSD on dissociation in BPD patients may be associated with the history of sexual abuse in childhood [38, 39]. Our finding that significantly more BPD patients with PTSD reported having attempted suicide than patients without PTSD diagnosis is consistent with the results of several previous studies [4, 8]. When controlling for CSA, the connection between PTSD and the history of suicide attempts disappeared, suggesting that CSA and not PTSD, is influencing suicidality rates in BPD patients. Similar previous results have been reported stating that severity of CSA is associated with higher rates of suicide attempts [19, 20]. For example, Ferraz and colleagues revealed that in BPD patients, the presence, number and severity of previous suicide attempts are significantly predicted by CSA [40]. In recent studies [8, 13] it has been assumed that CSA could be associated with the effects of PTSD in BPD patients and even account for the presence of PTSD symptoms among patients with BPD [18]. Interestingly, BPD patients with and without PTSD did not differ in terms of the frequency of suicide attempts. However, our findings indicate that the frequency of suicide attempts was significantly related to CSA.

While self-mutilation tended to be higher in BPD patients with co-occurring PTSD, this trend was not statistically significant, despite the relatively large number of patients included in our study. This is not fully consistent with previous studies, which revealed that BPD patients with co-occurring PTSD had a higher frequency of intentional self-injury, typically being triggered by flashbacks, nightmares or thoughts about sexual abuse or rape [13, 14]. The slight differences between our finding and previous results might relate to the very high prevalence of self-mutilation in our study (>90 % in both subsamples of BPD patients with and without PTSD), which might have caused a ceiling effect. Self-mutilation was unrelated to CSA in our sample.

With respect to the impact of PTSD on BPD severity, our study provided preliminary evidence that some specific aspects (rather than overall severity) might be impacted by co-occurring PTSD. With respect to overall severity, we only found a non-significant trend towards higher total scores in the BSL. We found no evidence for higher scores with respect to the number of BPD criteria and the dimensional IPDE score in the subgroup of patients with co-occurring PTSD. This is in line with previous reports that PTSD does not necessarily affect the extent of BPD psychopathology [11, 13, 18]. With respect to the facets of psychopathology (BSL), we did, however, detect higher scores in the subscales “intrusion” and “affect regulation” in BPD patients with co-occurring PTSD. This indicates that patients with both diagnoses report more intrusions and elevated problems in affect regulation than BPD patients without a co-occurring PTSD diagnosis. Overall, these results are in line with the findings by Harned and colleagues, who did not find an effect for PTSD on the number of BPD criteria and the dimensional score, but on emotion regulation [13]. However, these results do not support the outcomes of other studies, which indicated that co-occurring PTSD has a negative effect on general BPD symptomatology [8, 10, 14].

Our study has both strengths and limitations. A strength of our study is the relatively large sample of well diagnosed participants. All patients underwent standardized diagnostics including structured interviews for BPD (IPDE) and Axis-I disorders (SCID-I), which were conducted by experienced diagnosticians. With respect to the limitations, we would like to emphasize that our sample only included treatment-seeking female BPD patients recruited at specialized university settings. Hence, generalizations beyond the population investigated in this study should be made with caution. Furthermore, as most of our dependent variables were based on retrospective self-reports, there is a possibility of bias. One significant limitation concerns the assessment of the presence and frequency of previous suicide attempts and the history of self-mutilation in this study. As we did not use a well-established and validated measure, the validity of our results on suicide attempts and self-mutilation might be constrained. Future studies investigating this topic should make use of measures such as the Columbia-Suicide Severity Rating Scale (C-SSRS), which have been developed to consistently define and classify these behaviours and show good psychometric properties [41]. Furthermore, as we conducted an observational study, the possibility of making any causal conclusions regarding the impact of PTSD and CSA in BPD patients is precluded. Finally, multiple testing poses the question regarding the inflation of alpha-error. To address this issue, a Bonferroni-correction was applied. We found that dissociation and a history of at least one suicide attempt would still hold after correction for multiple testing.

## Conclusions

Despite these limitations, the results of our study should be considered in further research, as well as in clinical practice. From an etiological perspective, it is interesting that both dissociation and a history of at least one suicide attempt were related to CSA rather than to PTSD. From a clinical perspective, our data highlights the need to address these aspects in the treatment of BPD patients with co-occurring PTSD or a history of CSA, as suicide attempts are highly prevalent within this population of patients and as dissociation has been shown to detrimentally affect the success of treatment in both BPD [13, 14] and PTSD patients [3].

## Abbreviations

BPD, borderline personality disorder; BSL, borderline symptoms list- 95; CSA, childhood sexual abuse; C-SSRS, Columbia-Suicide Severity Rating Scale; CTQ, childhood trauma-questionnaire; DES, dissociative experiences scale; GAF, global assessment of functioning scale; IPDE, International Personality Disorder Examination; PTSD, posttraumatic stress disorder; SCID-I, structural clinical interview for DSM-IV; SPSS, statistical package for the social sciences

## Footnotes

1 The mentioned percentages refer to 93 of the 142 patients with PTSD. For the remaining 49 patients information regarding the type of trauma (from SCID-I) was either missing or not entirely conclusive retrospectively.
